# Retinoid Agonists in the Targeting of Heterotopic Ossification

**DOI:** 10.3390/cells10113245

**Published:** 2021-11-19

**Authors:** Robert J. Pignolo, Maurizio Pacifici

**Affiliations:** 1 Department of Medicine, Mayo Clinic, Rochester, MN 55905, USA; 2 Translational Research Program in Pediatric Orthopedics, Children’s Hospital of Philadelphia, Philadelphia, PA 19104, USA; pacificim@chop.edu

**Keywords:** retinoids, retinoic acid receptors, chondrogenesis, heterotopic ossification, palovarotene

## Abstract

Retinoids are metabolic derivatives of vitamin A and regulate the function of many tissues and organs both prenatally and postnatally. Active retinoids, such as *all trans*-retinoic acid, are produced in the cytoplasm and then interact with nuclear retinoic acid receptors (RARs) to up-regulate the transcription of target genes. The RARs can also interact with target gene response elements in the absence of retinoids and exert a transcriptional repression function. Studies from several labs, including ours, showed that chondrogenic cell differentiation and cartilage maturation require (i) the absence of retinoid signaling and (ii) the repression function by unliganded RARs. These and related insights led to the proposition that synthetic retinoid agonists could thus represent pharmacological agents to inhibit heterotopic ossification (HO), a process that recapitulates developmental skeletogenesis and involves chondrogenesis, cartilage maturation, and endochondral ossification. One form of HO is acquired and is caused by injury, and another severe and often fatal form of it is genetic and occurs in patients with fibrodysplasia ossificans progressiva (FOP). Mouse models of FOP bearing mutant ACVR1R206H, characteristic of most FOP patients, were used to test the ability of the retinoid agonists selective for RARα and RARγ against spontaneous and injury-induced HO. The RARγ agonists were found to be most effective, and one such compound, palovarotene, was selected for testing in FOP patients. The safety and effectiveness data from recent and ongoing phase II and phase III clinical trials support the notion that palovarotene may represent a disease-modifying treatment for patients with FOP. The post hoc analyses showed substantial efficacy but also revealed side effects and complications, including premature growth plate closure in some patients. Skeletally immature patients will need to be carefully weighed in any future regulatory indications of palovarotene as an important therapeutic option in FOP.

## 1. Introduction

**Figure 1:**
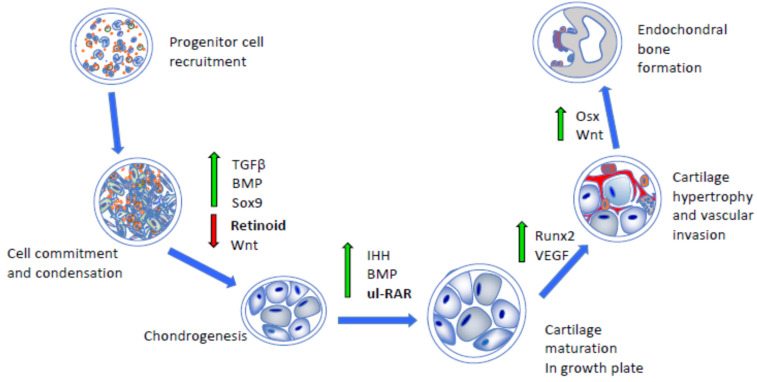
Schematic depicting the major developmental steps during endochondral bone formation. Following progenitor cell commitment and condensation (left panels), chondrogenic differentiation, cartilage maturation, hypertrophy, and vascular invasion require the concerted and stage-dependent up-regulation of indicated pathways and transcription factors (middle panels, green arrows). This leads to replacement of hypertrophic cartilage with bone (upright panel). Importantly, chondrogenesis and cartilage maturation require down-regulation of antagonistic pathways, such as **retinoid** and Wnt signaling (red arrow), as well as action by unliganded RNA receptors exerting transcriptional repression (**ul-RAR**). TGFβ, transforming growth factor β; BMP, bone morphogenetic protein; IHH, Indian hedgehog; VEGF, vascular endothelial growth factor; Osx, osterix.

The retinoids are metabolic derivatives of vitamin A, encompass a group of lipophilic molecules that include retinol, retinyl esters, and retinoic acid, and are essential for the embryonic development and postnatal functioning of multiple tissues and organs [1,2,3,4]. The skeleton is one such organ system strictly dependent on retinoids as classic studies carried out decades ago first demonstrated [5,6,7]. In humans, vitamin A is exclusively obtained from nutrition in the form of provitamin A carotenoids or other precursor molecules that are absorbed in the gut, metabolized by the intestinal mucosa, and transported to, and stored in, the liver and adipose tissues mostly as retinyl esters [8]. Upon systemic needs, these molecules are hydrolyzed and metabolized to retinol that is released into the circulation ferried by retinol-binding protein (RBP) [9]. The largely inactive retinol is taken up by cells in target tissues via the cell surface receptor and transporter STRAT6 [10]. Within the cytoplasm, sequential enzymatic action by retinol dehydrogenases (RDHs) and retinaldehyde dehydrogenases (RALDHs) converts the retinol into biologically-active *all*-*trans* retinoic acid (*at*RA) (Figure 1) [8]. Other active retinoids, such as *9*-*cis* RA and *13*-*cis* RA, can be produced, albeit at lower levels and in certain tissues only [11]. The resulting biologically active retinoids are transported to the nucleus by cellular retinoid binding proteins (CRABPs) where they associate with and regulate the transcriptional activity of retinoic acid receptors (RARs) and retinoid X receptors (RXRs) [12,13,14]. These receptors are normally present as heterodimeric RAR–RXR complexes that are associated with retinoic acid response elements (RAREs) located in the enhancer regions of target genes. The RAREs consist of two hexameric direct repeats (DRs) of the sequence (A/G)G(T/G)TCA separated by two or five base pairs (DR2 and DR5). These DR configurations elicit RAR–RXR binding specificity since hexameric repeats separated by four or three nucleotides (DR4 and DR3) act instead as response elements for thyroid hormone receptor/RXR complexes and vitamin D receptor/RXR complexes, respectively [13,15,16]. This selective regulatory property is known as the 3-4-5 rule [13]. Notably, the cellular levels of *at*RA and other active retinoids differ substantially amongst tissues and organs, including the cartilaginous growth plate, and can also be modulated at different stages of development or in response to specific needs [17,18]. Such levels are closely supervised and maintained by reciprocal levels of active retinoid-synthesizing enzymes (RDHs and RALDHs) versus retinoid–catabolic CYP26 enzymes [19,20,21]. The fundamental nature of these mechanisms is exemplified by the fact that the global ablation of the major RALDH isoform, *RALDH2*, has early embryonic lethality [22]. To fine-tune these regulatory loops further, *at*RA is able to regulate the expression of its own metabolic and receptor genes, including the *RARs*, *CYP26A1*, and carrier proteins [23,24], and can elicit autocrine and paracrine responses by producing and surrounding cells [25].

It is well recognized that heterodimeric RAR–RXR complexes interact and associate with RAREs even in the absence of active retinoids as it occurs in cells where cytoplasmic retinol is shunted to intracellular storage rather than processing into its active derivatives [13,25]. Importantly, these unliganded RAR–RXR heterodimers can elicit a transcriptional repression function in association with co-repressors, such as nuclear receptor co-repressors 1 and 2 (NCOR1 and 2), recruiting histone deacetylase and Polycomb complexes [26]. The availability and binding of active retinoids to the RAR–RXR heterodimers allow for protein conformational changes and the replacement of co-repressors with co-activators (NCOA1, 2, and 3) along with histone acetylases and other factors, leading to chromatin relaxation and the activation of target gene expression [13]. Transcriptional repression and activation are of fundamental importance and work in close concert to establish and regulate basic but essential tissue-specific gene expression patterns [27]. The ability of RAR complexes to switch from repressors to activators in response to local ligand availability and levels provides them with biological and developmental malleability and flexibility and can even be exploited therapeutically [28]. It is indeed an understanding of such basic RAR activator and repression roles during the skeletal development and growth that paved the way toward the creation of a retinoid agonist-based therapy against heterotopic ossification (HO) [29], which is elaborated upon below.

## 2. RARs, Retinoids, and Skeletal Development and Growth 

The RARs and retinoids are very ancient evolutionary mechanisms that first emerged in non-chordate animals [30]. In the vertebrate genome, there are three RAR genes (*RARα*, *RARβ*, and *RAR*γ) and three RXR genes (*RXRα*, *RXRβ*, and *RXR*γ) that are expressed in dynamic and differential spatio–temporal patterns [31,32]. The potential roles and biological significance of these genes in skeletal development and growth have been interrogated by the creation and analysis of single or compound mouse mutants. The global ablation of single *RAR* or *RXR* genes has been found to be well tolerated with the exception of *RXRα*, indicating that these receptors are characterized by significant functional redundancy [33]. However, this was not the case for compound mutant mouse embryos that displayed severe and pervasive defects in diverse organs and structures, including the developing skeleton. For example, compound *RARα*/*RAR*γ-null mouse embryos were shown to exhibit severe defects in their craniofacial, axial, and appendicular skeleton [34]. In the developing skull of the double mutants, several skeletal elements were markedly affected in the midface and cranial base. In the axial skeleton, there were homeotic transformations of several vertebrae, including the anteriorization of C2 and C5, displaying ectopic ribs and the malformations and misalignment of the vertebral bodies, neural arches, ribs, and sternum. Interestingly, the identity of each vertebra is under strict control by *Hox* genes [35], and several such genes are directly regulated by *at*RA signaling and action [36]. Thus, the homeotic transformation of vertebrae in the double RAR mutants probably resulted from altered patterns of *Hox* expression and/or shifts in the RAR transcriptional activation versus repressor function. In the appendicular skeleton of the double mutants, the defects were more severe in the forelimbs than the hindlimbs [34]. They included the fusion of several digits, loss of digit one in some mutants and polydactyly in others, delayed ossification, and sporadic absence of the radius and fibula. The exact causes of, and variability amongst, these changes remain unclear, but the overall severity of skeletal defects in mutants do point to specific and essential roles of RARs, RXRs, and retinoid action in early skeletal development.

Subsequent studies conducted by one of our laboratories focused on postnatal skeletal development and morphogenesis and, in particular, the possible expression and roles of RARs in the functioning of the cartilaginous growth plate [37,38], the key engine of skeletal elongation [39,40]. Typically, the growth plate is composed of resting, proliferative, pre-hypertrophic, and hypertrophic zones of chondrocyte maturation and is followed by the osseous primary spongiosa, thus permitting both skeletal elongation as well as endochondral bone formation up until the end of puberty when the growth plates close [39,40]. In juvenile mice, we observed that *RARα* and *RARβ* were expressed at low levels and in a diffuse manner in long bone growth plates, whereas *RAR*γ was very strongly expressed in the resting, proliferative, and pre-hypertrophic zones but not in the hypertrophic zone [38]. We created conditional mouse mutants deficient in *RAR* expression in cartilage by mating floxed *RAR* mice with *Col2*-*Cre* mice [41]. Compound *RARα*/*RAR*γ– or *RARβ*/*RAR*γ–deficient mice were characterized by significant growth retardation by 3 weeks of age, and their growth plates were abnormal and displayed scanty aggrecan, normally a prominent cartilage matrix component. To determine whether the RARs operate as unliganded or liganded receptors within the growth plate, we resorted to the direct quantification of endogenous retinoids using tissue microdissection and liquid chromatography/mass spectrometry (LC–MS/MS) analytical procedures [42]. Our data revealed that the growth plates are largely devoid of endogenous retinoids, providing evidence that the RARs are unliganded and operate as transcriptional repressors. The data were very much in line with previous studies showing that mouse long bone growth plates displayed negligible *RARE*-*LacZ* reporter activity [43] and strongly expressed the retinoid catabolic enzyme *CYP26B1* [44]. Thus, we interrogated multiple plausible transcriptional co-repressors and did find that *ZAC1* was very prominently expressed in the growth plate, and its expression overlapped that of *RAR*γ. Interestingly as well, the RARγ and ZAC1 proteins were able to physically interact with each other, and *ZAC1* over-expression in cultured chondrocytes under retinoid-free conditions enhanced chondrocyte phenotypic expression and functioning, likely supported by the increased expression of the cartilage master regulator *SOX9*.

Together, the studies above have provided clear evidence that the retinoids and RARs play important and multiple roles in prenatal skeletal development and postnatal growth and ossification, likely resulting from their ability to influence the expression of several target genes and modulate diverse cellular processes. The studies have established also that the growth plate is largely devoid of endogenous active retinoids, signifying that, under normal circumstances, the RARs operate as unliganded repressors to sustain its functioning (Figure 1). We should note that the ectopic tissue masses forming during HO initially display a fibroproliferative character and then undergo chondrogenesis and endochondral ossification [45,46,47], thus recapitulating the steps of normal skeletogenic processes and inspiring the creation of retinoid-based therapeutics based on those similarities, as described below.

## 3. Antagonistic Action of Retinoid Signaling on Chondrogenesis and Bone Morphogenetic Protein (BMP) Signaling 

At its onset during early embryogenesis, skeletal development starts with the formation of condensations of mesenchymal and ectomesenchymal stem cells that then commit to chondrogenesis, undergo differentiation, and lay down the initial cartilaginous blueprint of much of the future skeleton [48]. The chondrocytes become organized into growth plates to sustain growth and endochondral bone formation, as summarized above. Given the fundamental role of chondrogenesis in setting the skeletal development process in motion, there has been continuous and extensive interest for decades in its regulatory, cellular, and molecular mechanisms [39,40,49]. The regulation of chondrogenesis is also of central relevance to HO since this ectopic pathogenic process mandatorily requires chondrogenesis as well. An intriguing, key, and long-standing question in the field has been how the condensed mesenchymal cells commit and proceed toward chondrogenesis rather than undertake other developmental paths, such as fibrogenesis or adipogenesis. It has become clear that several pathways intervene to induce, facilitate, and promote the commitment to chondrogenesis, including *SOX5*/*6*/*9* genes and signaling by both BMP and transforming growth factor β (TGFβ family members (Figure 1) [40,50,51]. It has also become apparent that, concurrently, potential anti-chondrogenic pathways and mechanisms need to be shut down. One such prominent mechanism is the one regulated by Wnts and β-catenin signaling, which, if allowed to operate, would shunt the mesenchymal progenitors directly toward osteogenesis, bypassing chondrogenesis [51,52]. A second mechanism to be shut down is retinoid signaling and action (Figure 1) [53].

Interest in the retinoid biology and potential roles in early chondrogenesis was initially prompted by the finding that endogenous active retinoids, including *at*RA, were present in the form of an antero–posterior gradient across incipient early embryonic limb buds, indicating that the gradient may dictate differential cell determination and commitment to diverse cell lineages across the limb developmental field [54]. A subsequent study from one of our laboratories showed that *at*RA did differentially affect the progenitor cell subpopulations present within the limb bud [55]. It potently inhibited chondrogenesis, whose progenitors derive from the lateral mesoderm, but had no inhibitory effects on myogenesis, whose progenitors are somatic in origin [56]. The data related quite well with the findings using *RARE*-*LacZ* reporter mice in which active retinoid signaling was undetectable within limb mesenchymal cell condensations undergoing chondrogenesis and cartilage formation but was strong and prominent in the surrounding progenitors undergoing myogenesis and fibrogenesis [43]. Together, the studies affirmed the notion that chondrogenesis normally requires the absence of, or a major drop in, active retinoid signaling and action to start and proceed (Figure 1).

Subsequent important and revealing studies found that the transcriptional repression elicited by unliganded RAR receptors was actually required for chondrogenesis, as monitored by master chondrogenic gene expression and cartilage matrix production [57,58,59]. It was found also that *RARα* was initially expressed by early limb mesenchymal progenitors and was replaced by *RAR*γ expression as the cells committed and underwent chondrogenic differentiation. This switch was accompanied by a steep decrease in the *RALDH2* and *CRABP* expression and by a concurrent increase in *CYP26* expression, thus (i) depleting the early chondrogenic progenitors of endogenous retinoids and carrier proteins, (ii) repressing the anti-chondrogenic pathways by unliganded RARs, and (iii) favoring the expression and activity of pro-chondrogenic pathways, including BMP signaling. The latter mechanism was further explored in subsequent studies by comparing the global gene expression (over 360 genes) in limb bud progenitor cells under BMP stimulation toward chondrogenesis versus companion cells in which chondrogenesis was blocked by *at*RA [60]. The former cells displayed an up-regulation of canonical BMP signaling mediators, including phospho-SMAD1/5/8 proteins, and of pro-chondrogenic genes, including *DLX5*, *SOX9*/*5*/*6*, *ZAC1*, and *MATN3*, while the inhibition of chondrogenesis by *at*RA fully reversed those protein signaling and molecular pathways and promoted the resumption of endogenous active retinoid production and action by RALDH1A1, RALDHA2, RBP1, and CRABP2. Together, the above studies have provided further and clear evidence that active retinoid signaling is antithetical to the initiation and progression of chondrogenesis and exerts such a dampening role by suppressing pro-chondrogenic pathways, including BMP signaling, and by instigating anti-chondrogenic pathways at multiple levels. It is the penetrant and effective anti-chondrogenic ability of *at*RA that has inspired and paved the way for the testing of synthetic retinoid agonists for the treatment of HO [29], as described below.

## 4. Heterotopic Ossification and Pre-Clinical Studies on RARγ Agonists

HO encompasses the formation and accumulation of extraskeletal pathological bone at diverse anatomical sites. There is a common, acquired, and pathologically variable form of HO that is triggered by invasive surgeries, trauma, or deep burns and affects a large number of patients worldwide [29,45,61]. A second form of HO is congenital, rare, and severely debilitating and affects patients with fibrodysplasia ossificans progressiva (FOP;OMIM #135100) [46]. In FOP, HO is formed in the muscles, tendons, and ligaments and is often preceded by painful and recurrent exacerbations or flare-ups manifested as soft tissue swellings. Inflammatory episodes begin in early childhood and lead to progressive ankyloses of major joints with resultant immobility. The prognosis is poor, and the life expectancy is reduced.

Individuals with FOP appear normal at birth except for the malformation of the big toes, which are typically short and deviated in hallux valgus [46]. The HO formation is episodic and cumulative throughout life, resulting in segments, sheets, and ribbons of extra bone developing throughout the body and across joints, progressively restricting movement. Radiographic imaging and other evidence support that the process ultimately leading to new HO formation starts before clinical symptoms are reported. Asymmetric HO in the rib cage and or back in the presence of subsequent contralateral growth can lead to a rapid progression in spinal deformity and contributes to thoracic insufficiency syndrome, with cardio–respiratory failure and pneumonia the most common causes of death [62]. The ankyloses of the temporomandibular joints result in severe tooth decay and malnutrition. Most FOP patients are confined to a wheelchair by the third decade of life and require caregiver assistance to perform daily living activities. 

There are approximately 900 confirmed cases of FOP globally. The disease is inherited in an autosomal dominant fashion; however, due to low reproductive fitness, the disease is rarely transmitted through generations. Most patients afflicted with FOP have sporadic gain-of-function mutations in the BMP type I receptor (ACVR1), also known as ALK2 or activin-receptor-like kinase 2 [63]. Approximately 97% of all the known cases harbor the identical activating point mutation, a heterozygous single nucleotide substitution (a guanine to adenine change at position 617) that changes amino acid 206 from arginine to histidine (R206H mutation). Codon 206 is highly conserved and occurs within the glycine–serine (GS) region of the cytoplasmic domain of ACVR1 [63]. Additional mutations have been identified in both the GS domain and the kinase domain of the receptor with atypical forms of the disease [64,65,66,67,68]. The primary molecular pathology in FOP involves the BMP signaling pathway deranged by all the above mutations. 

Patients with FOP typically experience episodic flare-ups characterized by large painful swellings that can resolve spontaneously within weeks to months [69] but with most resulting in the formation of heterotopic bone. The signal that triggers spontaneous flare-ups is unknown, but the involvement of neurokinins has been postulated [70]. It is also well known that flare-ups can be induced by soft tissue injury, such as surgery, muscle fatigue or overstretching, intramuscular injections, and intramuscular immunizations. Viral illnesses can also induce flare-ups [46,71].

HO proceeds in two phases: a catabolic phase of inflammation and tissue destruction followed by an anabolic phase of tissue formation ultimately leading to mature heterotopic bone. The histological progression through these stages has been described in animal models of FOP and in misdiagnosed FOP patients through the collection of biopsied tissue samples taken at various stages of a flare-up [72]. The catabolic phase of HO is characterized by the perivascular infiltration of numerous lymphocytes, mast cells, and monocytes in connective tissue and skeletal muscle. The lymphocytes then expand into and around the muscle fibers with obvious muscle destruction [73]. The ensuing anabolic phase is initiated by the replacement of the degraded muscle tissue with copious fibroproliferative cells, followed by angiogenesis and vascularization [74]. Later in this phase, the skeletogenic progenitors undergo condensation and differentiation and produce a cartilage template, which undergoes maturation and hypertrophy and is subsequently replaced by endochondral bone (Figure 1). The HO that forms in the FOP is normal skeletal bone in terms of histology, biochemistry, metabolism, radiology, and biomechanics [74,75,76,77].

**Figure 2:**
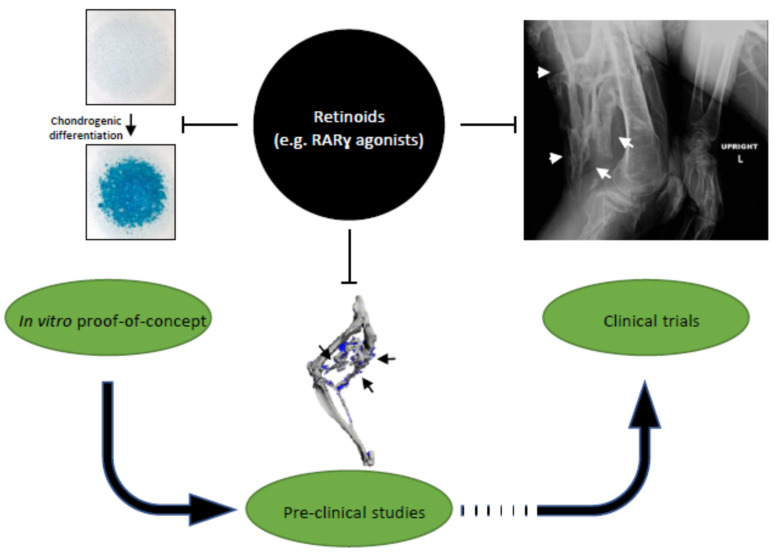
Translation of retinoid signaling roles in chondrogenic differentiation into clinical studies on inhibition of endochondral heterotopic ossification (HO). Retinoids, especially RARγ agonists, have inhibitory effects on chondrogenesis in micromass cultures (left); preclinical mouse models of HO (middle); and HO formation in fibrodysplasia ossificans progressiva (right).

Palovarotene is the brand name of 4-[(E)-2-(5,5,8,8-tetramethyl-3-pyrazol-1-ylmethyl-5,6,7,8-tetrahydronaphthalen-2-yl)-vinyl]-benzoic acid, which is an orally bioavailable RARγ-selective agonist and was originally known as R667 [78,79]. RARγ agonists at pharmacologic doses can potently impair skeletogenesis and endochondral ossification by redirecting prechondrogenic mesenchymal stem cells to a nonosseous soft tissue fate [80]. The rationale for testing them as inhibitors of HO in FOP [81] was based on the observation that retinoid signaling is a strong inhibitor of chondrogenesis [55] and that unliganded RAR transcriptional repressor activity is needed for chondrogenic differentiation (Figure 1 and Figure 2) [58,82]. The inhibition of HO with non-selective active retinoids or RARα receptor agonists has also been achieved but to a lesser degree [47,81]. RARγ agonists may exert their action on bone and cartilage through the post-translational regulation of BMP signaling by inhibiting Smad phosphorylation and promoting proteasome-regulated degradation of Smads specific to the BMP signaling pathway [81].

The palovarotene effectiveness has been evaluated in various animal models of HO, including a BMP-implant model, a constitutively active receptor model (Q207D), and a highly physiological human mutation knock-in model (R206H) [81,83]. Following injury, the results consistently demonstrate reductions in HO by palovarotene across the models, and a significant reduction in spontaneous (non-injury) HO with chronic treatment (Figure 2). It should be noted that inhibition of HO formation was shown to be a drug class effect that is shared by other retinoid agonists [81] and that there was dose-related inhibition of HO formation with higher doses of palovarotene. The effectiveness of the retinoids was the highest for RARγ-selective agonists as opposed to the pan-agonist *at*RA and an RARα-selective agonist. These studies suggested that both the effectiveness and sustainability of the inhibitory effect on HO formation were mediated via the RARγ receptor subtype. The effectiveness of RARγ selective agonists has also been shown to cover a wide treatment window that includes the prechondrogenic fibroproliferative phase up to, but not including, the ossification phase [81].

In addition to the injury-based models above, palovarotene has also been found to be effective in preventing HO formation in a spontaneous HO model [83]. The ACVR1 R206H knock-in mouse model limits the expression of the mutant allele (ACVR1 R206H) in the developing embryo to a population of skeletal progenitor cells (Prrx1+) and leads to HO formation in the absence of injury (Prrx1-R206H model). The Prrx1-R206H model recapitulates many of the phenotypic features of FOP seen in patients, including malformed big toes. An average human equivalent dose of approximately 5-mg of palovarotene administered every other day by oral gavage to young Prrx1-R206H mice substantially reduced the formation of spontaneous HO [83].

## 5. Target and Off-Target Effects 

As outlined above, the desired target effects of RARγ selective agonists relate to the inhibition of chondrogenic differentiation with the subsequent inhibition of HO. However, these same target effects in skeletally immature animals or humans could potentially lead to impaired physeal cartilage maturation/differentiation, causing premature growth plate closure and decreased bone growth. The threshold for dose-limiting skeletal toxicity is expected to increase with age.

The mucocutaneous effects of RARγ selective agonists are numerous [84]. Skin lesions related to the use of palovarotene include erythema, edema, epithelial hyperplasia, hyperkeratosis, and/or epidermal hypergranulosis across multiple species and in a dose-dependent manner. The squamous epithelium of other tissues, such as the non-glandular mucosa of the stomach, esophagus, and conjunctivae of the eye, may also be affected. In the FOP phase 2 and phase 3 studies, the most frequently reported adverse events were primarily mucocutaneous in nature, and the incidence of mucocutaneous and dermatologic events increased with increasing the palovarotene flare-up dose [85,86]. These events included cheilitis/dry lips, dry skin, pruritus, and alopecia (hair thinning). The events were generally mild or moderate in severity; however, skin and soft tissue infections were reported as severe and/or serious. Dose modifications (reductions) due to mucocutaneous events were required more frequently during flare-up dosing than during chronic dosing. Drug eruption was also commonly reported related to “retinoid dermatitis,” defined as a triad of generalized rash, dry skin, and erythema with the onset of all three findings within 2 to 3 days. The reproductive and developmental toxicity profile of palovarotene is generally consistent with what has been described for hypervitaminosis A and reported with other systemic retinoids [87]. Possible adverse events include testicular degeneration and teratogenicity. The weight of evidence suggests that palovarotene does not pose a genotoxic hazard to human subjects; however, no studies have been done to evaluate the carcinogenic hazard posed by palovarotene.

Musculoskeletal events, such as arthralgia, pain in extremities, and unspecified conditions aggravated, were also commonly reported. Subjects with open epiphyses who are enrolled in the phase 2 and phase 3 FOP palovarotene studies undergo knee and hand/wrist radiographs (anterior/posterior view) for the assessment of epiphyseal growth plates and linear and knee height measurements for the assessment of growth. At the pre-treatment baseline in the FOP studies, the most common epiphyseal growth plate abnormality was growth recovery lines (dense metaphyseal lines), followed by sclerosis of adjacent growing bone. Some subjects had new or worsening post-baseline growth recovery lines and new sclerosis on follow-up radiographs. Up to about one-third of skeletally immature subjects experienced premature physeal closure (PPC) [85,86]. These events were observed as early as the month-6 X-ray assessment, and most were seen by month 12. All but one subject had PPC first observed at the distal femoral growth plate. Further analysis also suggests palovarotene may negatively impact growth in subjects with premature closure. The adverse event profile of young adult subjects and older compared to teenage subjects and younger was generally similar except for PPC. Older subjects had a higher incidence of other adverse events related to an increased disease burden expected with age. It remains unclear why growth side effects were observed in some but not all of the patients receiving palovarotene and why some growth plates appeared to close prematurely but many did not.

## 6. Clinical Trials on the RARγ Agonist Palovarotene

As mentioned above, palovarotene is an orally bioavailable selective RARγ agonist under investigation for the treatment of FOP (Figure 2). There are four studies in the FOP palovarotene clinical development program that provide key insights into the efficacy and risk/benefit of palovarotene in affected individuals.

NCT02322255 was a prospective, protocol-specified, longitudinal natural history study (NHS) of FOP [88]. NCT02190747 was a randomized, double-blind, and placebo-controlled phase II trial [89], and NCT02279095 is its ongoing open-label extension [90]. These three studies were designed to allow for adaptations to the individual study designs. Based on emerging non-clinical and clinical data at the time, the dosing regimens were refined as the studies progressed. Flare-up and disease progression outcomes were assessed across all three studies. The HO incidence and volume were assessed annually by standardized low dose whole-body computed tomography (WBCT) and/or during flare-ups by flare-up body region CT. Other clinical, patient-reported, and exploratory outcomes were assessed. The safety was monitored throughout all studies, including the NHS. These three studies informed the design of the phase III MOVE trial (NCT03312634) [91].

The efficacy and safety of palovarotene were also evaluated in the phase III MOVE trial (NCT03312634) [91]. In this open-label trial, the annualized change in the whole-body volume of new HO in patients with FOP treated with the chronic/flare-up regimen was assessed and compared with data from the NHS. Although the pre-specified futility criteria were met in MOVE, the subsequent post hoc analysis showed substantial efficacy, as measured by the lower whole-body volume of new HO [85]. Eighteen-month interim analyses included 97 patients from MOVE and 98 NHS participants with post-baseline WBCT data. The mean annualized new HO volume was about 62% lower in MOVE versus the NHS (post hoc, non-square, root-transformed analysis) [85]. Almost 30% of the subjects reported ≥ 1 serious AE, including PPC)/epiphyseal disorder in patients who were skeletally immature at the baseline.

We await comparative efficacy data among the current interventional clinical trials involving palovarotene, activin A antibody (i.e., garetosmab), small molecule kinase inhibitors (i.e., saracatinib), and rapamycin, and, until then, it is difficult to balance the relative merits of the various therapeutic strategies. However, Pignolo & Kaplan have recently surveyed the potential targets and therapeutic interventions being considered in the drug development pipeline for FOP [92].

## 7. Concluding Remarks 

Experimental studies by several groups, including ours, have established the primacy and importance of retinoid signaling in regulating the behavior and development fate of progenitor cells during skeletal development and growth. It is thus clear that retinoid signaling needs to be kept minimal or altogether prevented for skeletogenic cells to commit to chondrogenesis and produce cartilage templates undergoing endochondral ossification and bone formation. It is this very notion and underlying mechanisms that provided the theoretical and factual basis of a retinoid agonist-based treatment against extraskeletal endochondral bone formation in FOP [29].

The translation of retinoid biology into clinically meaningful studies on safety and efficacy in FOP patients genetically predisposed to HO formation has occurred rapidly (Figure 2). A major action of RARγ agonists is to promote the degradation of the Smad proteins involved in the BMP signaling pathway that are over-activated by the ACVR1 R206H mutation, resulting in an overall down-regulation of BMP signaling in pre-chondrogenic cells. This is likely to be one major mechanism by which palovarotene reduces the trauma-induced and spontaneous HO in animal models of FOP as well as in patients. As we summarized above, encouraging results have in fact been obtained from the pooled post hoc data based on three clinical trials that have evaluated the efficacy of different palovarotene dosing regimens in preventing new HO in patients with FOP following a flare-up [85]. The results from this post hoc pooled analysis, along with those of the phase III MOVE trial, may support palovarotene as a disease-modifying treatment for patients with FOP. Although the post hoc analyses showed substantial efficacy, the PPC risk in skeletally immature patients must be carefully weighed in any future regulatory indications of palovarotene as an important therapeutic option in FOP.

## Data Availability

Not applicable.
